# Hippocampal Amyloid Burden with Downstream Fusiform Gyrus Atrophy Correlate with Face Matching Task Scores in Early Stage Alzheimer’s Disease

**DOI:** 10.3389/fnagi.2016.00145

**Published:** 2016-06-17

**Authors:** Ya-Ting Chang, Chi-Wei Huang, Nai-Ching Chen, Kun-Ju Lin, Shu-Hua Huang, Wen-Neng Chang, Shih-Wei Hsu, Che-Wei Hsu, Hsiu-Hui Chen, Chiung-Chih Chang

**Affiliations:** ^1^Departments of Neurology, Cognition and Aging Center, Kaohsiung Chang Gung Memorial Hospital, Chang Gung University College of MedicineKaohsiung, Taiwan; ^2^Department of Nuclear Medicine and Center for Advanced Molecular Imaging and Translation, Chang Gung Memorial HospitalTaoyuan, Taiwan; ^3^Department of Nuclear Medicine, Kaohsiung Chang Gung Memorial Hospital, Chang Gung University College of MedicineKaohsiung, Taiwan; ^4^Department of Radiology, Kaohsiung Chang Gung Memorial Hospital, Chang Gung University College of MedicineKaohsiung, Taiwan; ^5^Department of Physical Education, National Kaohsiung University of Applied ScienceKaohsiung, Taiwan

**Keywords:** Alzheimer’s disease, amyloid, AV-45 PET, fusiform gyrus, gray matter volumetry, visual network

## Abstract

**Purpose**: Neuronal activity during face matching shows co-activation of the fusiform gyrus (FG) and areas along the ventral visual network. To elucidate the mechanisms related to the facial discrimination deficits in Alzheimer’s disease (AD), the study evaluates the relationships between β-amyloid (Aβ) load and gray matter (GM) atrophy within the ventral visual network.

**Methods**: Comprehensive cognitive assessments and GM volumetry using 3-dimentional T1-weighted images and AV-45 positron emission tomography (PET) were studied in 44 patients with AD. We used AV-45 PET to measure regional Aβ to analyze the correlations between the regional neocortical AV-45 retention and atrophy in patients with AD.

**Results**: FG volume was positively correlated with the para-hippocampus (β = 0.565, *P* < 0.001), posterior cingulate cortex (PCC; β = 0.402, *P* < 0.001), and hippocampus volumes (β = 0.209, *P* = 0.044). After carefully confounded all possible factors simultaneously, the hippocampus standardized uptake value (SUV) ratio was independently associated with FG volume (β = −0.151, *P* = 0.017). Furthermore, volumes of the hippocampus (*r* = 0.473, *P* = 0.003), para-hippocampus (*r* = 0.515, *P* = 0.001), and FG (*r* = 0.383, *P* = 0.018) were associated with Benton’s facial recognition test (BFRT).

**Conclusions**: In conclusion, our study indicated that amyloid burden within the hippocampus might contribute to FG cortical hub GM atrophy. While the face matching task scores were related to the FG, hippocampus, and para-hippocampus volumes, concordant changes of the aforementioned three structures suggested the importance of the three ventral visual network hubs in AD.

## Introduction

Deficits in visual strategies development in Alzheimer’s disease (AD) can lead to visuospatial deficits of facial perception and location matching (Hargrave et al., 2002; Cronin-Golomb and Hof, 2004). The identification of face or objects requires a higher level of functional interactions among the regions belonging to two visual network systems. The ventral visual network system takes part in object or facial discrimination tasks (Haxby et al., 1991) that includes the lingual gyrus, lateral temporal cortex, hippocampus, para-hippocampus, inferior frontal gyrus (FG), and orbitofrontal gyrus (Macko et al., 1982; Mishkin et al., 1983; Puce et al., 1995; Ungerleider et al., 1998). Specifically, the ventral visual network is connected feed-forwardly and backwardly with FG, a cortical hub well known for facial perception (Teipel et al., 2007; Tsao et al., 2008). The dorsal visual network system, in contrast, participates in spatial working memory and location-matching with areas including the superior and inferior parietal lobe, angular, supramarginal gyrus, and dorsolateral prefrontal cortex (Macko et al., 1982; Mishkin et al., 1983; Ungerleider et al., 1998). Besides the two visual networks, the primary visual cortex (that is, the cuneus and calcarine gyrus) participates in spatial attention (Gandhi et al., 1999), while the anterior cingulate cortex (ACC) and posterior cingulate cortex (PCC) play roles in visual stimuli coordination (Hahn et al., 2007).

In AD, several studies have tested the upstream and downstream relationships of amyloid toxicity theory (Hardy and Selkoe, 2002), using *in vivo* neuroimaging modalities. Quantitative measures obtained from amyloid positron emission tomography (PET) can act as upstream biomarkers and those obtained from magnetic resonance imaging (MRI) can act as downstream neuronal injury markers. Studies have shown that higher amyloid burden may lead to volume reduction locally or distantly (Mormino et al., 2009; Bourgeat et al., 2010; Chetelat et al., 2010; Chang et al., 2015). A previous functional magnetic resonance imaging (fMRI)-based study suggested that the FG activation had positive correlations with gray matter (GM) densities of the ventral visual network hubs (Teipel et al., 2007). However, the upstream or downstream relationships with regards to the connectomes interactions are less clear because of the methodological design of the fMRI-based study. In AD, amyloid plaques and loss of synapses often co-occur in FG and within the ventral visual network regions (Lewis et al., 1987; Tsao et al., 2008). Using the aforementioned amyloid PET or MRI biomarkers, the pathological loads and degenerative processes within the visual network-associated connectomes can be tested. Based on the literature review, it is possible that the neuropathological loads in FG can lead to worsened facial or object recognition abilities via degeneration of interconnected ventral visual network connectomes.

Based on the amyloid toxicity theory, we investigated the inter-relationships of FG amyloid burden or FG-related neurodegeneration with the interconnected visual networks. The propagation of amyloid pathology along the ventral visual network provides an *in vivo* model to determine how atrophy and amyloid burden within FG was related to reduced interconnected GM density. As the amyloid deposition appears earlier in AD than GM atrophy (Jack et al., 2010), the upstream and downstream relationships among selected parameters can be tested in a cross-sectional manner. Within the ventral visual network (Teipel et al., 2007) and adjusted for confounding co-variants, we additionally tested the GM structural covariances from selected regions of interests.

## Materials and Methods

### Inclusion and Exclusion Criteria

Forty-four patients with AD were enrolled from the Cognition and Aging Center at the Department of Neurology of Chang Gung Memorial Hospital from 2011 to 2015. Subjects were included based on the consensus of panels that composed of neurologists, neuropsychologists, neuroradiologists, and experts in nuclear medicine (Huang et al., 2010; Huang C. W. et al., 2013). AD was diagnosed according to the International Working Group criteria (McKhann et al., 2011). Only those with a positive PET amyloid visual reading, as rated by the nuclear physician, were included. The study additionally included age-matched healthy controls; however, AV-45 PET was not conducted for them. The control data were used to delineate the significant visuospatial changes and regional atrophy in AD.

All the patients with AD received stable treatment with acetylcholine esterase inhibitors from the time of diagnosis. The exclusion criteria were clinical stroke history, modified Hachinski ischemic score >4 (Rosen et al., 1980), and depression.

### Study Design

The Human Ethics Committee of our hospital approved the study protocol, and all the participants or their authorized caregivers provided written informed consent. The cognitive testing, MRI, or AV-45 PET was performed within 4 weeks.

### MRI Acquisition and Cortical Volumetric Analysis

MRI images were acquired by using GE 3T Signa Excite scanner (GE Medical System, Milwaukee, WI, USA). The scanning protocol included: (1) fluid attenuated inversion recovery, turbo spin-echo sequence with repetition time (TR)/echo time (TE)/flip angle: 9000 ms/85 ms/180°, 240 × 240 mm field of view (FOV), 320 × 224 matrix, and 34 slices with a thickness of 4 mm were acquired in 2 min and 44 s; and (2) T1-weighted, inversion-recovery-prepared, three-dimensional, spoiled, gradient-recalled acquisition in a steady-state sequence with repetition time/inversion time of 8600 ms/450 ms, 240 × 240 mm FOV, and 1-mm slice thickness.

Using the Statistic Parametric Mapping software version 8^1^, the preprocessing of T1 MRI involved removal of non-relevant tissue, intensity and spatial normalization to the MNI space, and tissue segmentation. We used the Individual brain Atlases using Statistical Parametric Mapping (IBASPM)^2^ (Tzourio-Mazoyer et al., 2002; Dewey et al., 2010) for regional labeling and volumetric calculations. The regional labeling was identified after aligning to the 116 automatic anatomical label (AAL) structures and the volume of each identified structure was calculated using the IBASPM toolbox of *Volume Statistic*. Using *Segmentation* in IBASPM, the images were segmented into cerebrospinal fluid (CSF), GM, and white matter (WM). The raw volumes of interest (VOI) volume and total intracranial volume (TIV) were calculated based on the *Volume Statics* result from IBASPM. TIV represented the sum of GM, WM, and CSF volumes, and the VOI volumetric statistics were made controlled for TIV (Barnes et al., 2010).

Nine VOIs within the ventral visual network were defined that included the inferior frontal, orbitofrontal, superior, middle and inferior temporal gyrus, hippocampus, para-hippocampus, lingual, and FG. In addition, we selected four VOIs from the dorsal visual network, which included superior or inferior frontal and superior or inferior parietal gyrus. As the ACC and PCC were important hubs in AD and participated in visual processing tasks, they were additionally selected as VOIs.

### AV-45 PET Acquisition and Analysis

AV-45 was synthesized at the cyclotron facility of Chang Gung Memorial Hospital. PET acquisition protocol, optimal scanning time, and image reconstruction followed a previous study (Huang K. L. et al., 2013). In brief, helical computed tomographic (CT) images were obtained for attenuation correction at 40 min. Each PET acquisition consisted of two 5-min dynamic frames obtained 50 min post injection in three-dimensional (3D) mode by using Biograph mCT PET/CT system (Siemens Medical Solutions, Malvern, PA, USA). Summed images were subsequently created for further analysis.

PET images were first co-registered to the 3D T1-weighted images by non-linear transformation using Statistical Parametric Mapping version 8 software (Wellcome Trust Centre of Cognitive Neurology, University College London, London, UK). The global and regional GM AV-45 loads were defined by using the MRI GM-segmented images, and the cerebellar GM represented the reference region. The standardized uptake value (SUV) was related to the injection dose and was normalized to the body weight. The SUV ratio (SUVr) was calculated by determining the ratios of SUV between the global and regional GM and the reference cerebellar cortical region.

### Neuropsychological Assessments

A trained neuropsychologist conducted the following tests: mini-mental state examination (MMSE), Benton’s facial recognition test (BFRT; Benton et al., 1994), Rey-Osterrieth (R-O) complex copy (Boone, 2000), visual object and space perception (VOSP; Warrington and James, 1991), and Chinese version verbal learning test (CVVLT; Chang et al., 2010). For CVVLT, the 10 min recall score (CVVLT-10 min) was used to represent the short-term episodic memory ability.

### Statistical Analysis

All values were expressed as mean ± standard deviation (SD). The correlations between the continuous variables were analyzed by the Pearson’s correlation. If significant correlations existed, stepwise regression analysis was made to determine the most significant predictors that were adjusted for age, sex, and TIV. For structural co-variance, relationship between FG GM volume and the volume in other regions of visual network was tested using partial correlation test, adjusted for age, sex and TIV. The independent relationship of volume between FG and its correlated regions was determined using linear regression model. For pathological co-variance, relationship between FG AV-45 SUVr and AV-45 SUVr in other regions of visual network was tested using partial correlation test, adjusted for age and sex. The independent relationship of AV-45 SUVr between FG and its correlated regions was determined using linear regression model. All statistical analyses were conducted using the Statistical Package for Social Sciences software package (version 18 for Windows^®^, SPSS Inc., Chicago, IL, USA).

## Results

### Demographic and Clinical Characteristics

There were no significant differences between AD and control groups in age (AD, 71.5 ± 7.8 years; control, 71.0 ± 7.3 years), education level (AD, 9.3 ± 4.9; control, 8.8 ± 3.6), and R-O complex copy scores (AD, 15.7 ± 3.7; control, 15.8 ± 3.0; *P* > 0.05). Compared with controls, AD patients showed significantly lower scores for CVVLT-10 min (AD, 3.8 ± 3.1; control, 6.8 ± 1.9; *P* < 0.001), MMSE (AD, 23.1 ± 4.6; control, 27.3 ± 3.2; *P* < 0.001), BFRT (AD, 4.4 ± 1.8; control, 5.1 ± 1.2; *P* = 0.027), VOSP (AD, 6.7 ± 3.3; control, 8.0 ± 2.1; *P* = 0.032) and adjusted hippocampal volumes (AD, 0.0037 ± 0.0008; control, 0.0056 ± 0.0027; *P* < 0.001).

### Volumetric Analysis Between FG and Visual Network VOIs

**Figure 1:**
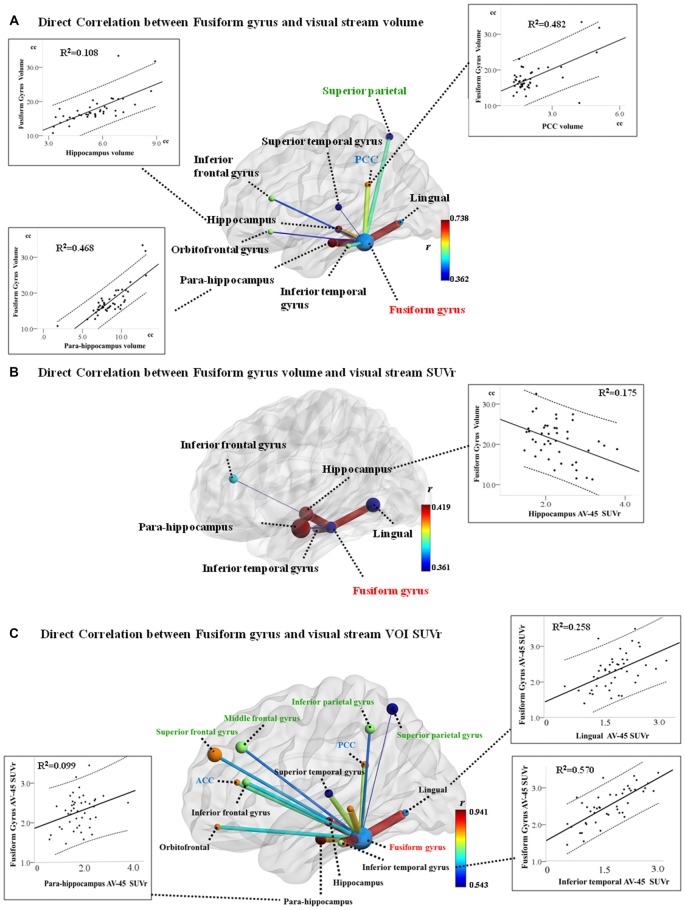
**Structural co-variance (A), volume-amyloid burden (B) and amyloid burden co-variance (C) between the fusiform gyrus (FG) and visual network hubs.** Model of the FG volume and structural covariant regional volume, and scatter plot of the age, sex, and total intracranial volume (TIV)-adjusted FG volumes with volumes of the hippocampus, para-hippocampus, and the posterior cingulate cortex (PCC). Superior parietal volume inversely correlates with FG volume exclusively. **(B)** Model of inverse correlation between FG volume and regional AV-45 standardized uptake value (SUV) ratio (SUVr), and scatter plot of the age, sex, and TIV-adjusted FG volume with SUVr in the hippocampus. **(C)** Model of positive correlation between the regions within visual stream and FG AV-45 SUVr, and scatter plot of the age and sex-adjusted FG SUVr with SUVr in the inferior temporal, lingual gyrus, and para-hippocampus. (Dotted lines represent 95% confidence limits for linear regression; regions with black text belong to the ventral visual stream; regions with green text belong to the dorsal visual stream).

Significant correlation between GM volume of FG and VOIs of dorsal visual network was found in the superior parietal volume (*r* = −0.528, *P* < 0.001). In addition, significant correlation between ventral visual network VOI and FG are shown in Figure 1A. After fitting all the aforementioned VOIs (showing structural covariance with FG) into the multiple regression model, only the hippocampus (β = 0.209, *P* = 0.044), para-hippocampus (β = 0.565, *P* < 0.001), and PCC volumes (β = 0.402, *P* < 0.001) were independently associated with FG volume (Figure 1A, scattered plots).

### FG Volume and Amyloid Burden within the Visual Network VOIs

Significant relationships between four ventral visual network VOI SUVr and FG volume are shown in Figure 1B. After fitting all the aforementioned four VOIs into the regression model, only the hippocampus SUVr (Figure 1B, scattered plots) showed statistical significance.

### SUVr Analysis Between FG and Visual Network VOIs

Direct correlations showed significant relationships between SUVr values of FG and all VOI, after adjusting for age and sex (*P* < 0.05; Figure 1C). In multiple regression analyses, only the inferior temporal (β = 0.557, *P* < 0.001), lingual gyrus (β = 0.329, *P* = 0.001), and para-hippocampus (β = 0.144, *P* = 0.047) showed independent role for FG SUVr (Scatter plots). There was a lack of correlation between FG SUVr and any of the VOIs volume (*P* > 0.05).

### Associations Between Visuospatial Function Scores and Amyloid Burden or Regional Volume in AD

After making adjustments for age, educational level, and sex, the BFRT scores correlated with volumes of the ACC (*r* = 0.387, *P* = 0.016), hippocampus (*r* = 0.473, *P* = 0.003), para-hippocampus (*r* = 0.515, *P* = 0.001), lingual (*r* = 0.354, *P* = 0.029), and FG (*r* = 0.383, *P* = 0.018). In addition, the lingual volume correlated with VOSP (*r* = 0.354, *P* = 0.029) and R-O complex copy (*r* = 0.393, *P* = 0.015).

## Discussion

### Main Findings

In mild stage AD, this study tested the upstream and downstream relationships between regional amyloid burden and GM volume within the visual networks, with the visuospatial scores as the major outcome measure. We have three major findings. The first was that we identified significant covariance between FG and the visual network VOIs in GM volume or SUVr. Interestingly, the relationships were not parallel in volume and amyloid. In volume co-variance analysis, significance was found in the para-hippocampus, PCC, and hippocampus while the lingual, inferior temporal, and para-hippocampus amyloid showed co-variance with the FG. The second, based on the amyloid theory, our study suggested that hippocampal AV-45 SUVr was related inversely to FG volume that further determined the scores in BFRT. Lastly, while no correlation was found between FG SUVr and visual network VOI volume, our results suggested that the pathological burden within the ventral visual network may be an upstream event leading to FG degeneration that determined the facial matching score.

### Upstream Ventral Visual Network Amyloid Burden Leads to FG Degeneration

The amyloid toxicity theory suggested that the accumulation of amyloid can be the primary driving force of neurodegeneration (Hardy and Selkoe, 2002; Masters et al., 2006) and is independent of tau tangles pathology (Lim et al., 2014). The influence of amyloid toxicity can be seen in regions that were distantly located, for example, the default mode networks (Chang et al., 2015) or the inferior temporal amyloid burden with hippocampal volume (Bourgeat et al., 2010). Within the visual network, our study showed association between the hippocampal AV-45 SUVr and FG volume. As no relationship was found between FG AV-45 SUVr and hippocampal volume in this study, the upstream hippocampus pathological burden and downstream neurodegeneration in FG is proposed.

Based on our study, we suggested that hippocampal amyloid burden may also show adverse effects on FG volume. Hippocampus is highly responsive to emotional facial expression stimuli and is important in regulating visual network regions activation (Brooks et al., 2012). Additionally, the importance of the hippocampus has been established by the event-related potentials experiment (Henson, 2015). As one of the critical hubs in the ventral visual network, the hippocampus neurons may send out a top-down signal to the FG that modulates the responses in repetitive facial stimulation test (Henson, 2015). From the biological perspectives, the projection fibers to the FG are sent by the descending pathways from the hippocampus (Gilbert and Li, 2013).

### Structural Covariance Between FG and Visual Network

In fMRI studies, spatial similarities between structural covariance network and intrinsic functional networks were established (Seeley et al., 2009). While using the FG as a seed in this study, the GM spatial co-variance were significant in ventral visual network hubs, the PCC, and superior parietal region. The structural covariance between the FG with most of the ventral visual hubs, but not the dorsal ones, may reflect the intrinsic functional networking of the ventral visual network (Bokde et al., 2006). In addition, our regression model showed the clinical weighting of two ventral visual hubs, which were the para-hippocampus and hippocampus. As the para-hippocampus, hippocampus, and FG were all part of the ventral visual networks, similar degrees of volume reduction can be expected (He et al., 2007).

A significant inverse relationship in GM volume was found between the superior parietal region and FG, although no significance was observed after fitting into the regression model. In patients with mild cognitive impairment, similar inverse relationships were found in the FG and GM volumes along the dorsal visual networks (Bokde et al., 2006; Teipel et al., 2007). Whether the paradoxical result represents a compensatory mechanism still need to be established. However, the differences of FG volume changes with respect to the dorsal or ventral visual networks supported the different functional networks involved in visual processing (Seeley et al., 2009).

Besides the visual network hubs, our study showed the volume relationships between the FG and PCC. The PCC is one of the key hubs of the default mode networks and represents early densely amyloid-affected regions whether in patients with AD (Li et al., 2008) or in cognitively normal subjects with a positive family history of AD (Mosconi et al., 2010). In postmortem study, the concentration of amyloid β (A β) in the PCC GM strongly correlated with episodic memory impairment (Pivtoraiko et al., 2015). The PCC is known for its role of modulating information that arrives FG through the interregional connection (Vogt et al., 2006), and it is implicated in facial expression processing. As the PCC and FG have strong connection, similar degrees of volume reduction can also be expected (Palop et al., 2006).

Aside from the structural co-variance between the FG and the ventral visual network VOIs, our study also found pathological co-variance between the FG and lingual, para-hippocampus, and inferior temporal gyrus. In comparisons, regions showing spatial patterns of amyloid loads were not parallel to the volumetric analysis. As the aforementioned four VOIs represent hubs within the ventral temporal cortex (Haxby et al., 2001), the pathological co-variance is speculated to be related to the regional adjacency (Haxby et al., 2001). Based on the data of fMRI, the four VOIs are also the strongest connected regions among the ventral visual networks (Haxby et al., 2001), simultaneously responding to viewing pictures of faces and object categories including houses, chairs, scissors, shoes, and bottles (Haxby et al., 2001). Coherence spatial pattern of amyloid deposition and intrinsic functional connectivity were reported before (Myers et al., 2014). Pathological co-variance pattern in this study suggested a mechanistic link between the amyloid deposition and intrinsic connectivity.

### Neurodegeneration in the Visual Network Predicts Different Visuospatial Tasks

Structure-cognitive analysis in this study has identified that the volumes of the FG, hippocampus, para-hippocampus, ACC, and lingual gyrus were linked with BFRT task scores. The findings were consistent with the previous literature of the FG, hippocampus, para-hippocampus, lingual gyrus, and ACC in facial matching task (Bokde et al., 2006). Although the ACC was not considered a part of the ventral visual network (Macko et al., 1982; Mishkin et al., 1983; Puce et al., 1995; Ungerleider et al., 1998), the ACC-PCC axis is one of the key components of default mode network (Greicius et al., 2004). In addition to the direct relationship, the influence might be via the PCC through the ACC-PCC connection (Vogt et al., 2006).

Similar to the results of another study (Melrose et al., 2013), the lingual gyrus volume was the only region that showed significance for VOSP location and R-O complex copy scores prediction. Additionally, lingual gyrus is activated by all kinds of visual stimulation (Macaluso et al., 2000). Similar to R-O complex copy, VOSP emphasized space information (Boone, 2000; Quental et al., 2013). Both metabolism and GM atrophy studies provide the evidence of lingual gyrus neurodegeneration affecting the processing of visual attention (Melrose et al., 2013).

### Limitation

There are several limitations of this study. First, we only selected patients with mild stage AD, which might limit the theory to a selected population. Second, the conclusion with regards to the upstream and downstream relationships between hippocampus and visual network hubs were based on the theory of amyloid toxicity. This does not imply that all of these factors (i.e., VOI volume, VOI SUVr and cognitive test) are inter-related. The effect of VOI volume on face-match test scores may be entirely independent of the effect of regional amyloid on volume. Third, noise signal from the WM region might overestimate the cortical AV-45 SUVr. During the stage of GM segmentation, however, the overlapping regions of cortical gyration and the VOI SUVr were carefully rechecked to avoid influence from the WM signals.

## Conclusion

In conclusion, our study indicates that amyloid burden within the hippocampus may contribute to FG cortical hub GM atrophy. While the face matching task scores were related to the FG, hippocampus and para-hippocampus volumes, concordant changes of the aforementioned three structures suggested the importance of the ventral visual network hubs in AD.

## Author Contributions

Y-TC participated in the design of the study, drafted the manuscript and performed the statistical analysis. Chi-WH, N-CC, K-JL, S-HH, W-NC, S-WH, Che-WH and H-HC participated in the sequence alignment, clinical evaluation of patients and helped draft the manuscript. C-CC helped draft the work and revise it critically for important intellectual content. All authors read and approved the final manuscript.

## Conflict of Interest Statement

The authors declare that the research was conducted in the absence of any commercial or financial relationships that could be construed as a potential conflict of interest.

## Footnotes

^1^http://www.fil.ion.ucl.ac.uk/spm/software/

^2^http://www.thomaskoenig.ch/Lester/ibaspm.htm
